# Metaplastic Effects of Ketamine and MK-801 on Glutamate Receptors Expression in Rat Medial Prefrontal Cortex and Hippocampus

**DOI:** 10.1007/s12035-021-02352-7

**Published:** 2021-03-15

**Authors:** Alessandro Piva, Lucia Caffino, Francesca Mottarlini, Nicholas Pintori, Fernando Castillo Díaz, Fabio Fumagalli, Cristiano Chiamulera

**Affiliations:** 1 Neuropsychopharmacology Lab, Section Pharmacology, Department Diagnostic & Public Health, University of Verona, Policlinico GB Rossi, P.le Scuro 10, 37134 Verona, Italy; 2 Department of Pharmacological and Biomolecular Sciences, University of Milano, Via Balzaretti 9, 20133 Milano, Italy

**Keywords:** Metaplasticity, Ketamine, MK-801, Medial prefrontal cortex, Hippocampus

## Abstract

Ketamine and MK-801 by blocking NMDA receptors may induce reinforcing effects as well as schizophrenia-like symptoms. Recent results showed that ketamine can also effectively reverse depressive signs in patients’ refractory to standard therapies. This evidence clearly points to the need of characterization of effects of these NMDARs antagonists on relevant brain areas for mood disorders. The aim of the present study was to investigate the molecular changes occurring at glutamatergic synapses 24 h after ketamine or MK-801 treatment in the rat medial prefrontal cortex (mPFC) and hippocampus (Hipp). In particular, we analyzed the levels of the glutamate transporter-1 (GLT-1), NMDA receptors, AMPA receptors subunits, and related scaffolding proteins. In the homogenate, we found a general decrease of protein levels, whereas their changes in the post-synaptic density were more complex. In fact, ketamine in the mPFC decreased the level of GLT-1 and increased the level of GluN2B, GluA1, GluA2, and scaffolding proteins, likely indicating a pattern of enhanced excitability. On the other hand, MK-801 only induced sparse changes with apparently no correlation to functional modification. Differently from mPFC, in Hipp, both substances reduced or caused no changes of glutamate receptors and scaffolding proteins expression. Ketamine decreased NMDA receptors while increased AMPA receptors subunit ratios, an effect indicative of permissive metaplastic modulation; conversely, MK-801 only decreased the latter, possibly representing a blockade of further synaptic plasticity. Taken together, these findings indicate a fine tuning of glutamatergic synapses by ketamine compared to MK-801 both in the mPFC and Hipp.

**Supplementary Information:**

The online version contains supplementary material available at 10.1007/s12035-021-02352-7.

## Introduction

Several studies have shown that drugs of abuse may induce permissive changes that subsequently affect synaptic plasticity events [1], through a mechanism that has been defined as “*plasticity of synaptic plasticity*,” also called *metaplasticity* [2]. Metaplasticity has been observed at different cellular and behavioral levels and can be induced by modification of synaptic plasticity or neural assembly and connectivity [3] or by the exposure to environmental enrichment, stress, or drugs of abuse [4–6].

In addition, metaplasticity has been characterized as a process that contributes to render neural circuits more crystallized and less susceptible to remodeling [7]. Nowadays, however, little is still known about metaplasticity as a phenomenon contrasting relapse, e.g., facilitating extinction or acting against the effect of determinant factors promoting drug-seeking relapse. Finnie and Nader [8] proposed that the molecular events able to affect memory reactivation and reconsolidation act as metaplastic mechanisms. *N*-methyl-d-aspartate (NMDA) receptor antagonists such as MK-801 and ketamine have been shown to induce metaplastic changes of long-term potentiation (LTP) by facilitating tetanus-induced LTP in ex vivo hippocampal slices 24 h after treatment [9–11]. Single ketamine dosing has been shown to induce a cascade of molecular events leading to enhanced synaptic activity in brain areas involved in mood, motivation, and emotional memory (e.g., [12, 13]). On the other hand, repeated ketamine self-administration altered glutamate receptors expression and αCaMKII autophosphorylation when observed 24 h after the last infusion in the medial prefrontal cortex (mPFC), ventral striatum, and hippocampus (Hipp) [14, 15]. These ketamine effects are commonly defined in literature as metaplastic effects [10, 16, 17].

Recently, we showed that MK-801-induced metaplasticity was able to inhibit the reconsolidation of appetitive memory for the natural reward sucrose, together with a correlated increase of GluN2B, GluA1, and mGluR5 in NAc and GluN2B in the amygdala [18]. Moreover, we showed that acute ketamine given prior to context-induced relapse significantly inhibited responding, but no effects were observed on the reconsolidation of the contextual appetitive memory. The inhibition of renewal was correlated to decreased levels of GluA1 and mGluR5 in the Hipp, whereas the lack of effect on contextual memory reconsolidation was explained as resistance to destabilization due to decreased GluN2B expression [19].

In the present study, we aimed to further investigate MK-801 and ketamine effects on the expression of glutamate transporter-1 (GLT-1), glutamate receptors (GluRs), and related scaffolding proteins in the mPFC and Hipp when given under an acute dosing regimen known to induce metaplastic changes [9, 10]. Rats were treated with MK-801 or ketamine 24 h before the assessment of the expression of the GLT-1, NMDA receptor subunits GluN1, GluN2A, and GluN2B, the α-amino-3-hydroxy-5-methyl-4-isoxazolepropionic acid (AMPA) receptor subunits GluA1 and GluA2, and their related scaffolding proteins both in the whole homogenate and in the post-synaptic density.

## Material and Methods

### Animals

Subjects were 20 male Sprague-Dawley rats (Charles River, Italy) weighing 300 ± 30 g at the time of treatment. Rats were housed in pairs in temperature and humidity-controlled environment (19–23 °C, 60 ± 20%) on a 12-h light/dark cycle, with light ON at 7:30 p.m. Water and food were available ad libitum. Animals were treated during the dark phase of the light/dark cycle, and all the experimental procedures were carried out in accordance with the U.K. Animals (Scientific Procedures) Act of 1986 and associated guidelines and with EU Directive 2010/63/EU for animal experiments. All efforts were made to minimize animal suffering and to keep the lowest number of animals used.

### Pharmacological Treatment

At day 1, 4 groups of rats (5 rats/group) were injected with ketamine 10 mg/kg/mL i.v., saline solution 1 mL/kg i.v., MK-801 4 mg/kg/mL i.p., or saline solution 1 mL/kg i.p. The doses of MK-801 and ketamine used here were selected based on previous metaplastic evidence. In fact, 4 mg/kg i.p. MK-801 was able to facilitate the induction of LTP in ex vivo CA1-subiculum synapses 24 h after the treatment, while 10 mg/kg i.v. ketamine was able to enhance the magnitude of LTP in ex vivo Schaffer collateral-CA1 synapses, both in an electrophysiological setting [9, 10]. These doses were also reported to affect reward-related behavior in our studies [18, 19]. At day 2, 24 h after injection, rats were anesthetized with intraperitoneally 350 mg/kg/2 mL chloral hydrate (Fluka, Italy) and sacrificed. Brains were rapidly removed and medial prefrontal cortices (bregma + 3.20 mm; [20] and hippocampi (− 3.30 mm) were dissected from 2-mm-thick slices using a Coronal Brain Matrix (SouthPointe Surgical Supply, FL, USA). Tissues were immediately frozen on dry ice and stored at − 80 °C.

### Western Blot Assays

Proteins in the whole homogenate and post-synaptic density were analyzed as previously described with minor modifications [19]. Briefly, Hipp and mPFC were homogenized in a teflon-glass potter in cold 0.32 M sucrose buffer pH 7.4 containing 1 mM HEPES, 1 mM MgCl_2_, 1 mM NaHCO_3_, and 0.1 mM PMSF, in presence of commercial cocktails of protease (Roche, Monza, Italy) and phosphatase (Sigma-Aldrich, Milan, Italy). An aliquot of each homogenate was then sonicated and stored at – 20 °C. The remaining homogenate was centrifuged at 800×*g* for 5 min; the obtained supernatant was then centrifuged at 13,000×*g* for 15 min, obtaining a pellet. This pellet was resuspended in a buffer containing 75 mM KCl and 1% Triton X-100 and centrifuged at 100,000×*g* for 1 h. The resulting supernatant, referred as Triton X-100 soluble fraction (TSF, extra-synaptic fraction), was stored at − 20 °C; the pellet, referred as PSD or Triton X-100 insoluble fraction (TIF, post-synaptic density), was homogenized in a glass–glass potter in 20 mM HEPES, protease, and phosphatase inhibitors and stored at − 20 °C in presence of glycerol 30%. Total proteins have been measured in the homogenate and TIF fractions according to the Bradford Protein Assay procedure (Bio-Rad, Milan, Italy), using bovine serum albumin as calibration standard.

Equal amounts of proteins of the homogenate (10 μg) and of TIF fraction (8 μg) were run on a sodium dodecyl sulfate-8% polyacrylamide gel under reducing conditions and then electrophoretically transferred onto nitrocellulose membranes (GE Healthcare, Milan, Italy). Blots were blocked 1 h at room temperature with I-Block solution (Life Technologies Italia, Italy) in TBS 0.1% Tween-20 buffer and incubated with antibodies against the proteins of interest.

The conditions of the primary antibodies were the following: anti GLT-1 (1:5000, AbCam), anti GluN1 (1:1000, Invitrogen), anti GluN2B (1:1000, Cell Signaling Technology Inc.), anti GluN2A (1:1000, Cell Signaling Technology Inc.), anti SAP102 (1:1000, AbCam), anti GluA1 (1:1000, Cell Signaling Technology Inc.), anti GluA2 (1:2000, Cell Signaling Technology Inc.), anti SAP97 (1:1000, AbCam), anti PSD95 (1:2000, Cell Signaling Technology Inc.), and anti β-Actin (1:10000, Sigma-Aldrich). Results were standardized using β-actin as the control protein, which was detected by evaluating the band density at 43 kDa. Immunocomplexes were visualized by chemiluminescence using the Chemidoc MP Imaging System (Bio-Rad Laboratories). Gels were run 3 times each and the results represent the average from 3 different western blots, averaged and normalized by using a specific correction factor [21]. Uncropped immunoblots related to the protein expression levels measured in the whole homogenate and post-synaptic density of medial prefrontal cortex and hippocampus are presented in Online resource 1.

### Statistical Analysis

Data were collected in individual animals (independent determinations) and are presented as means and standard errors (SEM). Molecular changes produced by pharmacological treatment were analyzed by unpaired Student’s *t*-test. Prism 8.0 (GraphPad) was used to analyze all the data. Statistical significance was assumed at *p* < 0.05.

## Results

### Effects of Pharmacological Intervention on Medial Prefrontal Cortex Protein Levels

**Fig. 1:**
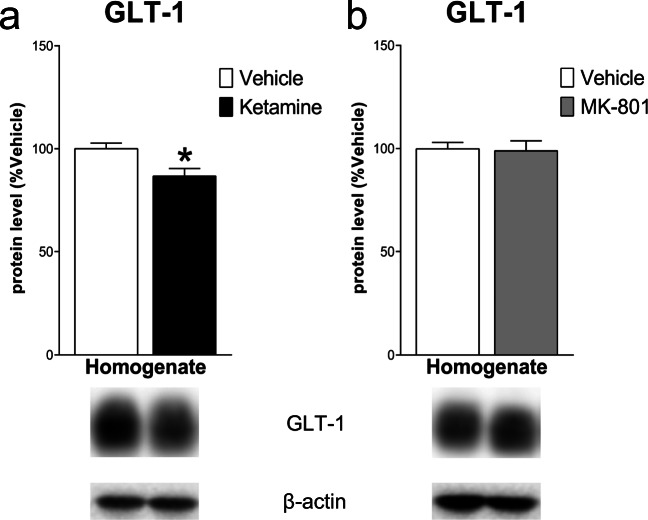
Effect of ketamine and MK-801 on glutamate transporter-1 (GLT-1) protein level in the total homogenate of mPFC 24 h after pharmacological treatments. Quantification of GLT-1 protein levels 24 after ketamine (**a**) or MK-801 (**b**) in the total homogenate. Below the graphs, representative immunoblots are shown for GLT-1 (62 kDa) and β-actin (43 kDa) proteins in the homogenate of mPFC. Data are shown as the mean + SEM and are expressed as percentage of the vehicle. Ketamine = solid column; MK-801 = grey column; vehicle = open columns. *N* = 5 rats/group. *p < 0.05 vs vehicle; unpaired two-tailed Student’s *t*-test

**Fig. 2:**
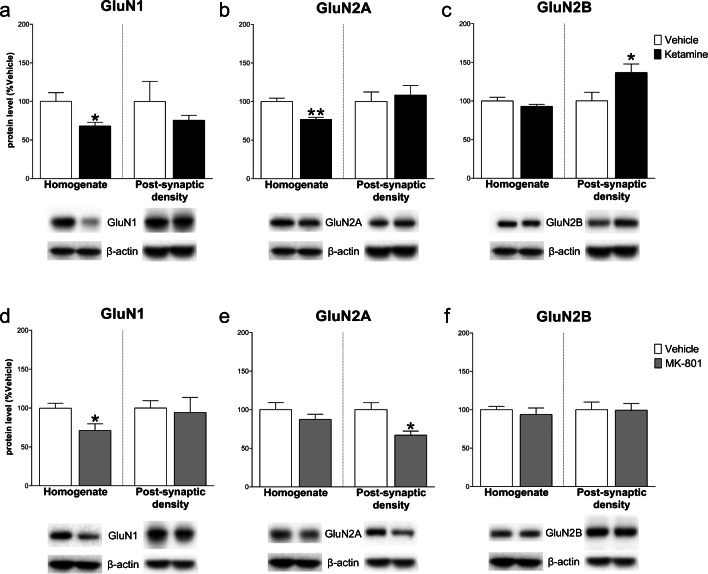
Effect of ketamine and MK-801 on NMDARs subunits in the homogenate and post-synaptic density of mPFC 24 h after pharmacological treatments. Quantification of GluN1 (**a**, **d**), GluN2A (**b**, **e**), and GluN2B (**c**, **f**) subunit protein levels 24 after ketamine (**top**) or MK-801 (**bottom**) in the total homogenate or in the post-synaptic density of mPFC. Below the graphs, representative immunoblots are shown for GluN1 (120 kDa), GluN2A (180 kDa), GluN2B (180 kDa), and β-actin (43 kDa) in the homogenate and in the post-synaptic density of mPFC. Data are shown as the mean + SEM and are expressed as percentage of the vehicle. Ketamine = solid columns; MK-801 = grey columns; vehicle = open columns. *N* = 5 rats/group. *p < 0.05, **p < 0.01 vs vehicle; unpaired two-tailed Student’s *t*-test

Western blot analysis in the mPFC 24 h after ketamine injection showed a significant decrease of GLT-1 protein levels in the total homogenate vs control (− 13.40 ± 4.7%, *t*_(8)_ = 2.879, *p* < .05, Student’s *t*-test); no significant changes were detected 24 h after MK-801 (− 1.00 ± 5.82, *t*_(8)_ = 0.172, NS) (Fig. 1). As far as concern NMDARs levels, in the total homogenate, ketamine decreased GluN1 and GluN2A subunit levels (respectively − 32.00 ± 12.40, *t*_(8)_ = 2.58, *p* < .05 and − 23.20 ± 5.25, *t*_(8)_ = 4.42, *p* < .01), with no changes for GluN2B (− 7.00 ± 5.49, *t*_(8)_ = 1.28, NS). On the other hand, analysis of the post-synaptic density revealed a significant increase of GluN2B (36.60 ± 15.83, *t*_(8)_ = 2.313, *p* < .05), with no changes for GluN1 and GluN2A (− 24.20 ± 26.91, *t*_(8)_ = 0.899 and 8.40 ± 17.78, *t*_(7)_ = 0.472; NS). Conversely, in the total homogenate, MK-801 injection only decreased GluN1 level (− 28.80 ± 10.78, *t*_(8)_ = 2.672, *p* < .05), with no effects on GluN2B and GluN2A (− 6.20 ± 9.49, *t*_(8)_ = 0.653 and − 12.60 ± 11.29, *t*_(8)_ = 1.116; NS). Similarly, no differences in the levels of GluN1 and GluN2B were observed in the post-synaptic density (− 5.8 ± 21.65, *t*_(8)_ = 0.268; − 0.60 ± 13.26, *t*_(8)_ = 0.045; NS) except for the decrease of GluN2A (− 33.00 ± 11.28, *t*_(7)_ = 2.925, *p* < .05) (Fig. 2).

**Fig. 3:**
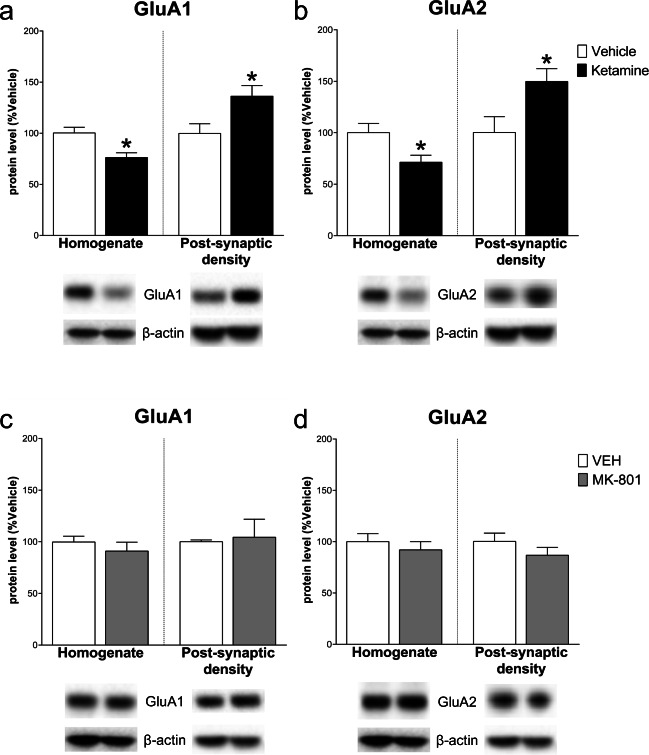
Effect of ketamine and MK-801 on AMPARs subunits in the homogenate and post-synaptic density of mPFC 24 h after pharmacological treatments. Quantification of GluA1 (**a**, **c**) and GluA2 (**b**, **d**) subunit protein levels 24 after ketamine (**top**) or MK-801 (**bottom**) in the total homogenate or in the post-synaptic density of mPFC. Below the graphs, representative immunoblots are shown for GluA1 (108 kDa), GluA2 (108 kDa), and β-actin (43 kDa) in the homogenate and in the post-synaptic density of mPFC. Data are shown as the mean + SEM and are expressed as percentage of the vehicle. Ketamine = solid columns; MK-801 = grey columns; vehicle = open columns. *N* = 5 rats/group. *p < 0.05 vs vehicle; unpaired two-tailed Student’s *t*-test

Regarding AMPARs subunit levels, while GluA1 and GluA2 levels decreased in the homogenate (− 24.00 ± 7.27, *t*_(8)_ = 3.304 and − 28.60 ± 11.26, *t*_(8)_ = 2.539; *p* < .05) and increased in the post-synaptic density vs control (36.45 ± 14.45, *t*_(7)_ = 2.522 and 49.60 ± 19.82, *t*_(8)_ = 2.503; *p* < .05) after ketamine, MK-801 did not induce any significant modulation, neither in the homogenate (GluA1: − 8.80 ± 10.33, *t*_(8)_ = 0.852; GluA2: − 8.00 ± 11.18, *t*_(8)_ = 0.715; NS) nor in the post-synaptic density (GluA1: 4.40 ± 17.50, *t*_(8)_ = 0.251; GluA2: − 13.40 ± 11.17, *t*_(8)_ = 1.200; NS) (Fig. 3).

**Fig. 4:**
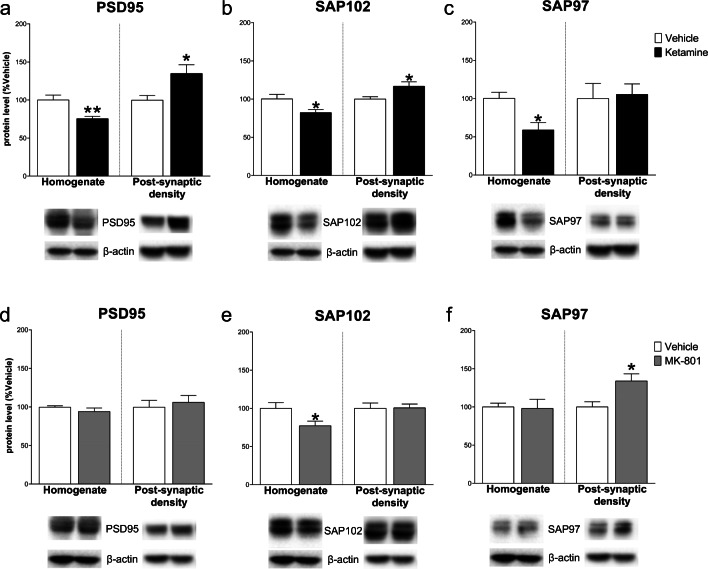
Effect of ketamine and MK-801 on scaffolding protein levels in the homogenate and post-synaptic density of mPFC 24 h after pharmacological treatments. Quantification of PSD95 (**a**, **d**), SAP102 (**b**, **e**), and SAP97 (**c**, **f**) subunit protein levels 24 after ketamine (**top**) or MK-801 (**bottom**) in the total homogenate or in the post-synaptic density of mPFC. Below the graphs, representative immunoblots are shown for PSD95 (95 kDa), SAP102 (102 kDa), SAP97 (97 kDa), and β-actin (43 kDa) in the homogenate and in the post-synaptic density of mPFC. Data are shown as the mean + SEM and are expressed as percentage of the vehicle. Ketamine = solid columns; MK-801 = dark grey columns; vehicle = open columns. *N* = 5 rats/group. *p < 0.05, **p < 0.01 vs vehicle; unpaired two-tailed Student’s *t*-test

Ketamine decreased the levels of scaffolding proteins PSD95, SAP102, and SAP97 in the homogenate (respectively, PSD95: − 24.60 ± 7.25, *t*_(8)_ = 3.393, *p* < .01; SAP102: − 18.00 ± 7.29, *t*_(8)_ = 2.468, *p* < .05; SAP97: − 41.40 ± 12.62, *t*_(8)_ = 3.279, *p* < .05), while increased both PSD95 and SAP102 in the post-synaptic density (PSD95: 35.00 ± 13.14, *t*_(8)_ = 2.663; SAP102: 16.80 ± 6.61, *t*_(8)_ = 2.543; *p* < .05; SAP97: 5.40 ± 24.06, *t*_(8)_ = 0.224, NS). On the other hand, the effect of MK-801 was less pronounced: in the homogenate, only SAP102 decreased compared to control (PSD95: − 5.80 ± 4.89, *t*_(8)_ = 1.185, NS; SAP102: − 22.80 ± 9.56, *t*_(8)_ = 2.386, *p* < .05; SAP97: − 2.00 ± 12.95, *t*_(8)_ = 0.154, NS), while only SAP97 increased in the post-synaptic density (PSD95: 6.00 ± 12.66, *t*_(8)_ = 0.474, NS; SAP102: 0.60 ± 8.57, *t*_(8)_ = 0.070, NS; SAP97: 34.00 ± 11.21, *t*_(7)_ = 3.034, *p* < .05) (Fig. 4).

**Fig. 5:**
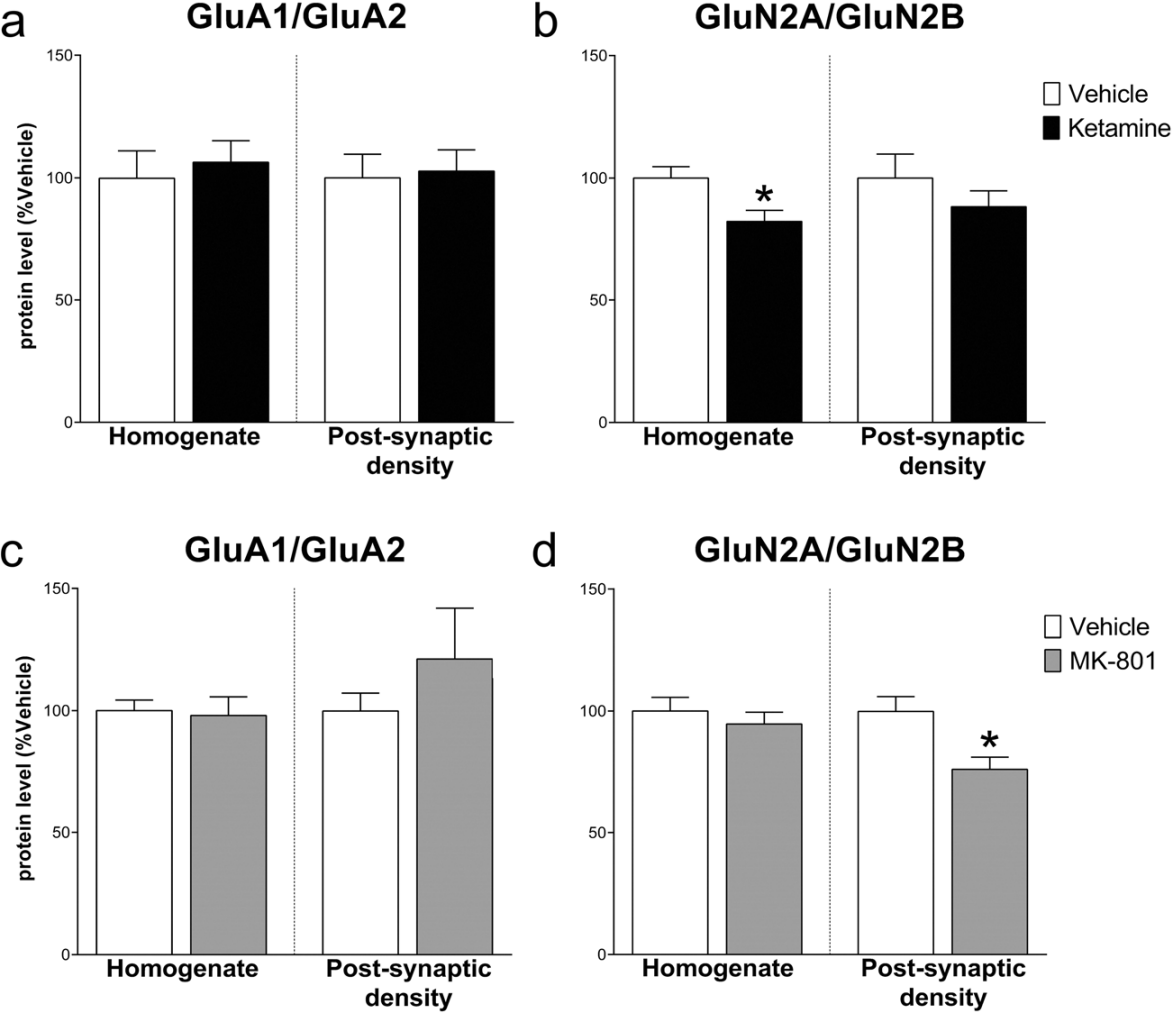
Effect of ketamine and MK-801 on AMPARs and NMDARs subunit ratios in the homogenate and post-synaptic density of mPFC 24 h after pharmacological treatments. Ratios of GluA1/GluA2 (**a**, **c**) and GluN2A/Glu2B (**b**, **d**) protein levels 24 after ketamine (**top**) or MK-801 (**bottom**) in the total homogenate or in the post-synaptic density of mPFC. Data are shown as the mean + SEM and are expressed as percentage of the vehicle. Ketamine = solid columns; MK-801 = dark grey columns; vehicle = open columns. *N* = 5 rats/group. *p < 0.05 vs vehicle; unpaired two-tailed Student’s *t*-test

As a functional correlate of synaptic plasticity and metaplasticity [8], we evaluated the ratio of GluN2A/GluN2B and GluA1/GluA2 in the mPFC. Analysis in the homogenate revealed a significant decrease of GluN2A/GluN2B after ketamine vs control (− 17.80 ± 6.52, *t*_(8)_ = 2.729, *p* < .05) but no changes in the post-synaptic density (− 11.80 ± 11.39, *t*_(7)_ = 1.036, NS). Conversely, MK-801 decreased the GluN2A/GluN2B ratio in the post-synaptic density (− 23.80 ± 7.86, *t*_(8)_ = 3.029, *p* < .05) but not in the homogenate (− 5.40 ± 7.44, *t*_(8)_ = 0.726, NS). Moreover, no changes resulted in the GluA1/GluA2 ratio, neither in the homogenate (ketamine: 6.60 ± 14.24, *t*_(8)_ = 0.464; MK-801: − 2.00 ± 8.75, *t*_(8)_ = 0.229; NS) nor in the post-synaptic density (ketamine: 2.80 ± 12.93, *t*_(7)_ = 0.217; MK-801: 21.40 ± 21.93, *t*_(8)_ =0.976; NS) (Fig. 5).

### Effects of Pharmacological Intervention on Hippocampal Protein Levels

**Fig. 6:**
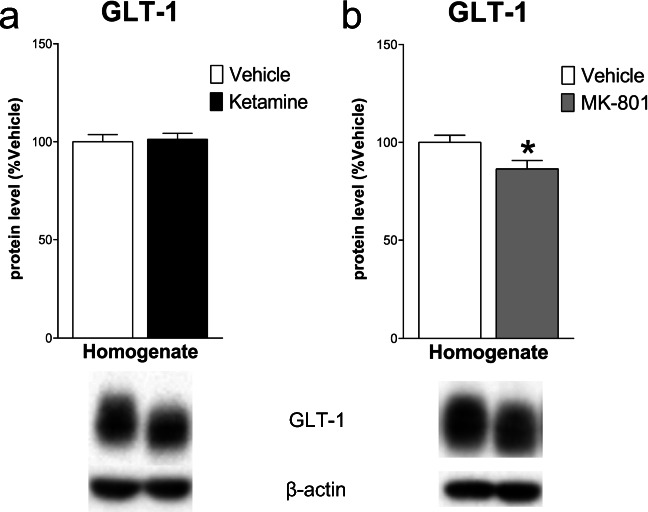
Effect of ketamine and MK-801 on GLT-1 protein level in the total homogenate of Hipp 24 h after pharmacological treatments. Quantification of GLT-1 subunit protein levels 24 after ketamine (**a**) or MK-801 (**b**) in the total homogenate. Below the graphs, representative immunoblots are shown for GLT-1 (62 kDa) and β-actin (43 kDa) proteins in the homogenate of Hipp. Data are shown as the mean + SEM and are expressed as percentage of the vehicle. Ketamine = solid column; MK-801 = grey column; vehicle = open columns. *N* = 5 rats/group. *p < 0.05 vs vehicle; unpaired two-tailed Student’s *t*-test

**Fig. 7:**
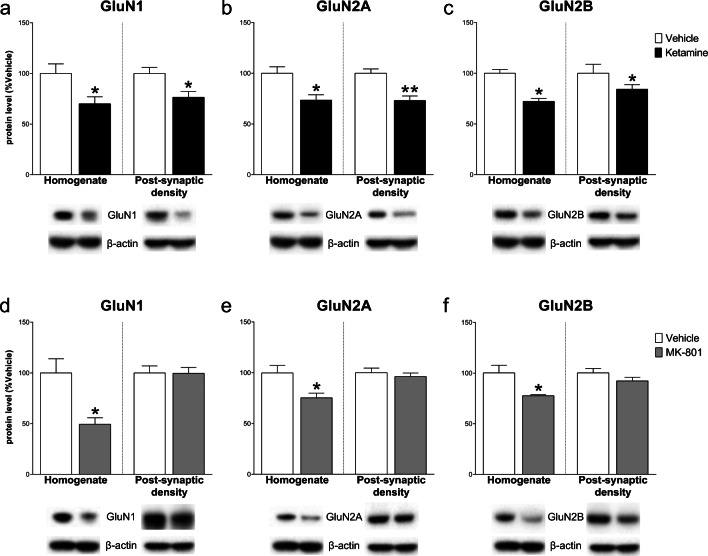
Effect of ketamine and MK-801 on NMDARs subunits in the homogenate and post-synaptic density of Hipp 24 h after pharmacological treatments. Quantification of GluN1 (**a**, **d**), GluN2A (**b**, **e**), and GluN2B (**c**, **f**) subunit protein levels 24 after ketamine (**top**) or MK-801 (**bottom**) in the total homogenate or in the post-synaptic density of Hipp. Below the graphs, representative immunoblots are shown for GluN1 (120 kDa), GluN2A (180 kDa), GluN2B (180 kDa), and β-actin (43 kDa) in the homogenate and in the post-synaptic density of Hipp. Data are shown as the mean + SEM and are expressed as percentage of the vehicle. Ketamine = solid columns; MK-801 = grey columns; vehicle = open columns. *N* = 5 rats/group. *p < 0.05, **p < 0.01 vs vehicle; unpaired two-tailed Student’s *t*-test

Analyses in the Hipp 24 h after pharmacological treatments revealed a significant decrease of GLT-1 levels after MK-801 (− 13.60 ± 5.63, *t*_(8)_ = 2.417, *p* < .05), but no changes after ketamine (1.20 ± 4.79, *t*_(8)_ = 0.251, NS) (Fig. 6). All NMDARs subunit levels decreased both in total homogenate (GluN1: − 30.00 ± 11.63, *t*_(8)_ = 2.579; GluN2A: − 26.60 ± 8.26, *t*_(8)_ = 3.222; GluN2B: − 27.80 ± 9.22, *t*_(8)_ = 3.015; *p* < .05) and in the post-synaptic density (GluN1: − 23.60 ± 8.17, *t*_(8)_ = 2.891, *p* < .05; GluN2A: − 27.00 ± 6.23, *t*_(8)_ = 4.335, *p* < .01; GluN2B: − 15.80 ± 5.84, *t*_(8)_ = 2.704, *p* < .05) after ketamine injection. MK-801 decreased NMDAR subunit levels in the homogenate (GluN1: − 50.60 ± 15.24, *t*_(8)_ = 3.319; GluN2A: − 24.60 ± 8.76, *t*_(8)_ = 2.809; GluN2B: − 22.60 ± 7.53, *t*_(8)_ = 3.001; *p* < .05) with no effects in the post-synaptic density (GluN1: − 0.40 ± 8.90, *t*_(8)_ = 0.045; GluN2A: − 3.80 ± 5.69, *t*_(8)_ = 0.668; GluN2B: − 8.00 ± 5.54, *t*_(8)_ = 1.444; NS) (Fig. 7).

**Fig. 8:**
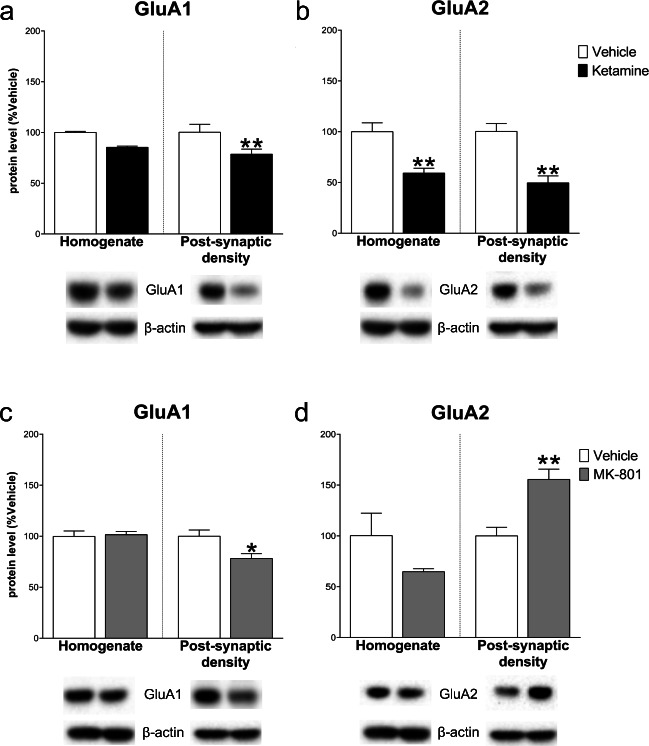
Effect of ketamine and MK-801 on AMPARs subunits in the homogenate and post-synaptic density of Hipp 24 h after pharmacological treatments. Quantification of GluA1 (**a**, **c**) and GluA2 (**b**, **d**) subunit protein levels 24 after ketamine (**top**) or MK-801 (**bottom**) in the total homogenate or in the post-synaptic density of Hipp. Below the graphs, representative immunoblots are shown for GluA1 (108 kDa), GluA2 (108 kDa), and β-actin (43 kDa) in the homogenate and in the post-synaptic density of Hipp. Data are shown as the mean + SEM and are expressed as percentage of the vehicle. Ketamine = solid columns; MK-801 = grey columns; vehicle = open columns. *N* = 5 rats/group. *p < 0.05, **p < 0.01 vs vehicle; unpaired two-tailed Student’s *t*-test

Ketamine and MK-801 modulated in a similar way GluA1 protein levels, which decreased only in the post-synaptic density both after ketamine (− 21.40 ± 5.11, *t*_(8)_ = 4.192, *p* < .01; homogenate: − 14.80 ± 7.97, *t*_(8)_ = 1.857, NS) and MK-801 (− 21.80 ± 7.72, *t*_(8)_ = 2.823, *p* < .05; homogenate: 1.60 ± 6.24, *t*_(8)_ = 0.257; NS). Conversely, GluA2 decreased both in the homogenate (− 40.80 ± 9.94, *t*_(8)_ = 4.10, *p* < .01) and in the post-synaptic density (− 50.60 ± 10.48, *t*_(8)_ = 4.829, *p* < .01) after ketamine, whereas MK-801 induced no effects in the homogenate (− 35.40 ± 22.30, *t*_(8)_ = 1.587; NS) and a significant increase in the post-synaptic density (GluA2: 55.60 ± 13.20, *t*_(8)_ = 4.213, *p* < .01) (Fig. 8).

**Fig. 9:**
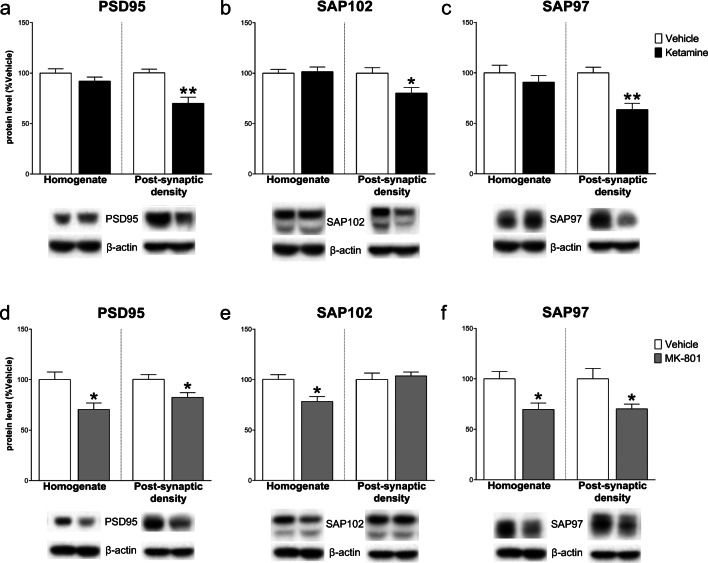
Effect of ketamine and MK-801 on scaffolding protein levels in the homogenate and post-synaptic density of Hipp 24 h after pharmacological treatments. Quantification of PSD95 (**a**, **d**), SAP102 (**b**, **e**), and SAP97 (**c**, **f**) subunit protein levels 24 after ketamine (**top**) or MK-801 (**bottom**) in the total homogenate or in the post-synaptic density of Hipp. Below the graphs, representative immunoblots are shown for PSD95 (95 kDa), SAP102 (102 kDa), SAP97 (97 kDa), and β-actin (43 kDa) in the homogenate and in the post-synaptic density of Hipp. Data are shown as the mean + SEM and are expressed as percentage of the vehicle. Ketamine = solid columns; MK-801 = dark grey columns; vehicle = open columns. *N* = 5 rats/group. *p < 0.05, **p < 0.01 vs vehicle; unpaired two-tailed Student’s *t*-test

Finally, scaffolding protein levels were decreased in the post-synaptic density (PSD95: − 30.20 ± 7.10, *t*_(8)_ = 4.256, *p* < .01; SAP102: − 19.80 ± 7.70, *t*_(8)_ = 2.573, *p* < .05; SAP97: − 36.40 ± 8.34, *t*_(8)_ = 4.364, *p* < .05) with no effects in the homogenate (PSD95: − 8.00 ± 5.85, *t*_(8)_ = 1.368; SAP102: 1.40 ± 6.00, *t*_(8)_ = 0.234; SAP97: − 9.20 ± 9.94, *t*_(8)_ = 0.925; NS) after ketamine. On the other hand, MK-801 decreased PSD95 and SAP97 both in the homogenate and post-synaptic density (PSD95: homogenate: − 29.60 ± 9.95, *t*_(8)_ = 2.974, post-synaptic density: − 17.80 ± 6.69, *t*_(8)_ = 2.659; *p* < .05; SAP97: homogenate: − 30.40 ± 9.69, *t*_(8)_ = 3.138, post-synaptic density: − 29.80 ± 11.36, *t*_(8)_ = 2.623; *p* < .05). SAP102 only decreased in the homogenate (− 22.00 ± 6.86, *t*_(8)_ = 3.206, *p* < .05; post-synaptic density: 3.60 ± 7.57, *t*_(8)_ = 0.475, NS) (Fig. 9).

**Fig. 10:**
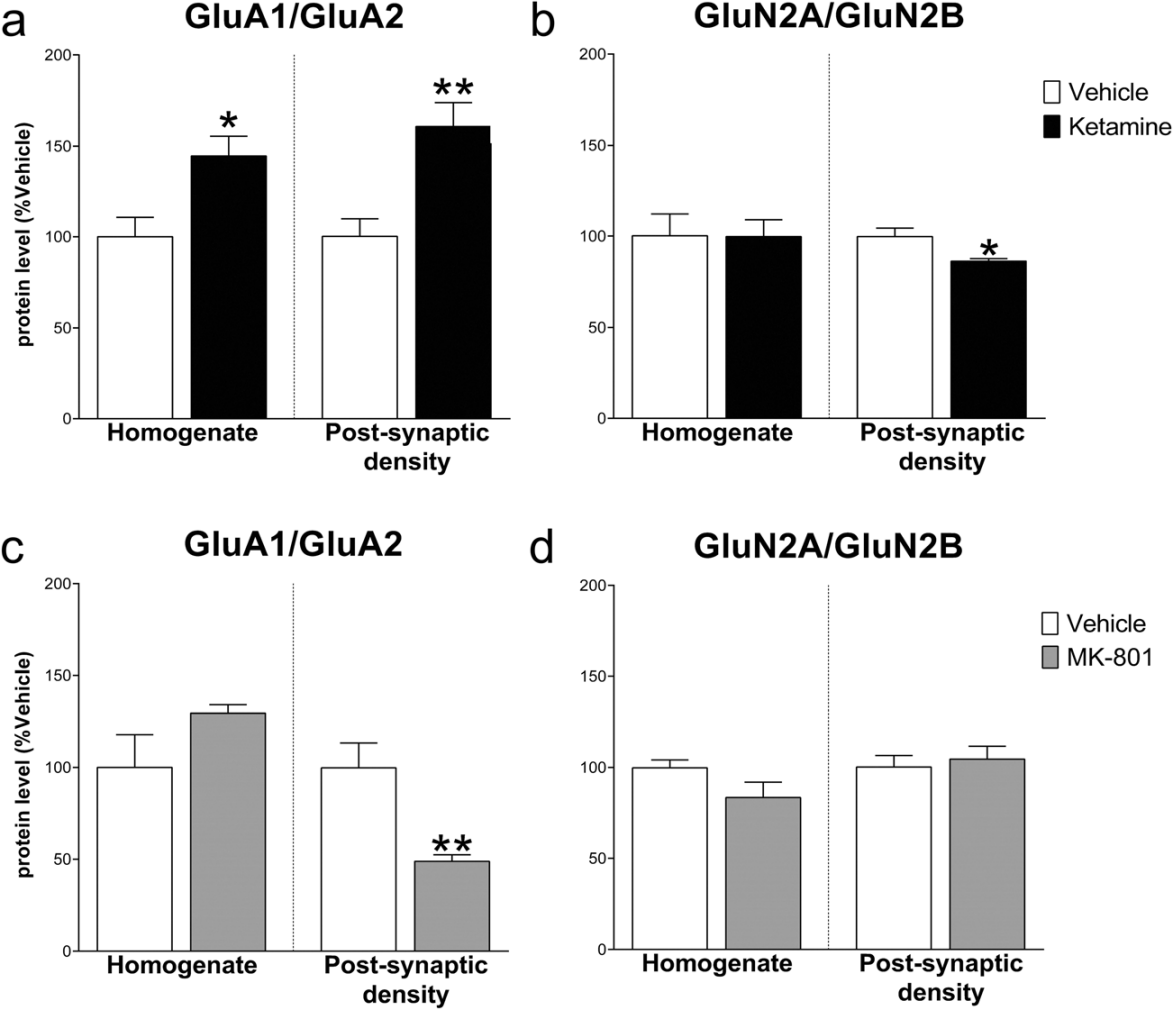
Effect of ketamine and MK-801 on AMPARs and NMDARs subunit ratios in the homogenate and post-synaptic density of Hipp 24 h after pharmacological treatments. Ratios of GluA1/GluA2 (**a**, **c**) and GluN2A/Glu2B (**b**, **d**) protein levels 24 after ketamine (**top**) or MK-801 (**bottom**) in the total homogenate or in the post-synaptic density of Hipp. Data are shown as the mean + SEM and are expressed as percentage of the vehicle. Ketamine = solid columns; MK-801 = dark grey columns; vehicle = open columns. *N* = 5 rats/group. *p < 0.05, **p < 0.01 vs vehicle; unpaired two-tailed Student’s *t*-test

Evaluation of GluN2A/GluN2B and GluA1/GluA2 ratios revealed different treatment effects: ketamine increased GluA1/GluA2 ratio at both homogenate (44.60 ± 15.28; *t*_(8)_ = 2.920, *p* < .05) and post-synaptic density level (60.60 ± 16.24, *t*_(8)_ = 3.733, *p* < .01), while decreased the GluN2A/GluN2B ratio only at the post-synaptic density level (− 13.60 ± 4.84, *t*_(8)_ = 2.813, *p* < .05; homogenate: − 0.40 ± 15.15, *t*_(8)_ = 0.026, NS). On the other hand, MK-801 only decreased GluA1/GluA2 ratio in the post-synaptic density (− 51.00 ± 13.96, *t*_(8)_ = 3.653, *p* < .01; homogenate: 29.60 ± 18.45, *t*_(8)_ = 1.604, NS) with no significant effects on GluN2A/GluN2B ratio vs control (homogenate: − 16.40 ± 9.54, *t*_(8)_ = 1.719; post-synaptic density: 4.40 ± 9.46, *t*_(8)_ = 0.465; NS) (Fig. 10).

## Discussion

Our results show that ketamine and MK-801 differently modulate the homeostasis of the glutamate synapse in the mPFC and Hipp 24 h after acute treatment.

In the mPFC, ketamine induced remarkable changes compared to MK-801; in fact, except for GluN2B, in the total homogenate, all the molecular targets analyzed were decreased after ketamine compared to vehicle. Interestingly, ketamine injection induced an opposite effect in the post-synaptic density. MK-801 only decreased the obligatory NMDAR subunit GluN1 and the scaffolding protein SAP102 in the homogenate. In the post-synaptic density, MK-801 decreased GluN2A subunit and increased SAP97 scaffolding protein. The GluN2A/GluN2B ratio, previously indicated as metaplastic correlate [8], showed an increase in the homogenate after ketamine and in the post-synaptic density after MK-801.

In the Hipp, ketamine decreased the level of all NMDARs subunits and of AMPAR subunit GluA2, whereas MK-801 significantly decreased glutamate transporter-1, all NMDARs subunits, and all scaffolding protein levels in the total homogenate. In the post-synapses, ketamine decreased the post-synaptic density levels of all NMDARs, AMPARs, and scaffolding proteins, while MK-801 increased GluA2 and decreased GluA1, PSD95, and SAP97 levels. GluA1/GluA2 and GluN2A/GluN2B ratios showed opposite changes after ketamine or MK-801: ketamine increased GluA1/GluA2 ratio both in the homogenate and in the post-synaptic density, while decreased GluN2A/GluN2B ratio in the post-synapses. On the other hand, MK-801 only decreases the GluA1/GluA2 ratio in the post-synaptic density. A summary of ketamine and MK-801 effects in the post-synaptic density of mPFC and Hipp is reported in Table 1.

**Table 1:** Summary table of ketamine and MK-801 effects on glutamate transmission components evaluated in the post-synaptic density of mPFC and Hipp 24 h after treatment

mPFC			Hipp		
Ket		MK-801	Ket		MK-801
↓	GLT-1	=	=	GLT-1	↓
=	GluN2A	↓	↓	GluN2A	=
↑	GluN2B	=	↓	GluN2B	=
=	GluN2A/GluN2B ratio	↓	↓	GluN2A/GluN2B ratio	=
↑	GluA1	=	↓	GluA1	↓
↑	GluA2	=	↓	GluA2	↑
=	GluA1/GluA2 ratio	=	↑	GluA1/GluA2 ratio	↓
↑	PSD95	=	↑	PSD95	=
↑	SAP102	=	↓	SAP102	=
=	SAP97	↑	↓	SAP97	↓

Symbols: ↑ = significant increase; ↓ = significant decrease; 0 = no change vs vehicle

The doses of MK-801 and ketamine herein used have been previously shown to induce metaplasticity in electrophysiological settings; in fact, systemic injection of 4 mg/kg MK-801 facilitated LTP induction following subthreshold high-frequency stimulation in ex vivo CA1-subiculum synapses 24 h after the treatment [9]. Similarly, 10 mg/kg intravenous ketamine increased the magnitude of LTP induced by submaximal high-frequency stimulation in ex vivo hippocampal slices 24 h after injection and increased Ca^2+^-permeable subunits GluA1 and GluN2B at the cell surface in medial PFC and Hipp [10]. Ketamine facilitation of LTP 24 h post-dosing was also confirmed at subcutaneous 30 mg/kg and for intravenous 3 mg/kg, with the latter facilitating LTP even at 72 h post-dosing [22]. More recently, we showed that this MK-801 dose administrated 24 h before sucrose instrumental memory reactivation was able to inhibit responding at reinstatement test, an effect correlated to glutamate receptors alterations in the nucleus accumbens and amygdala [18]. On the other hand, ketamine 24 h before contextual memory reactivation did not significantly affect the reconsolidation of sucrose contextual memory at reinstatement test, while significantly inhibited sucrose-seeking behaviors when administrated 24 h before relapse. Similarly, this ketamine dose induced glutamate receptors alterations in the nucleus accumbens and amygdala [19]. Thus, considering that these effects occurred long after the half-life of both the NMDARs antagonists in rats, i.e., approximately 1 h for ketamine and 2 h for MK-801 [23–25], our results further support the occurrence of metaplastic effects of both compounds on the glutamate synapse.

### The Effects of Ketamine and MK-801 in the Medial Prefrontal Cortex

Recently, we demonstrated that ketamine markedly reduced the level of glutamate receptors and related scaffolding proteins in the crude synaptosomal fraction of mPFC, Hipp, and ventral striatum after long-term self-administration [14, 15]. The general decrease of glutamate receptors and scaffolding proteins in the mPFC homogenate that we observed in the present study after acute administration could be related, instead, to reduced gene expression or protein translation or to increased protein turnover, similarly to reports on PCP effects [26] and as seen in schizophrenic and depressed patients [27, 28]. This decrease could potentially be mediated by the glutamate excess in the synaptic cleft due to the decreased level of the glutamate transporter GLT-1. However, in contrast with this hypothesis, Burgdorf et al. [10] showed no differences in the levels of mRNA transcripts for GluN2B and GluA1 in mPFC. The same authors reported also an increase of total cellular protein level for GluN2B and no differences for GluA1, partially in contrast with our results, i.e., no changes for GluN2B and a decrease for GluA1 in the homogenate.

The post-synaptic density profile of glutamate receptors and scaffolding proteins markedly differed from the homogenate: the increase of GluN2B subunit, as well as GluA1 and GluA2 subunits, indicates a general state of prefrontal cortex activation, in line with the disinhibition hypothesis of ketamine antidepressant action [29]. Moreover, the post-synaptic density levels of GluN2B and GluA1 in mPFC are in line with the cell surface results reported by Burgdorf et al. [10].

Interestingly, the ketamine-induced effect on GluN2B was paralleled by an increase in its scaffolding proteins PSD95 and SAP102. The level of PSD95 was shown to be decreased in both depressed subjects and animal models of depression [28], and this decrease was shown to be attenuated by acute ketamine [30, 31]. Moreover, a decrease in SAP102-enconding transcripts was detected in the striatum of major depressed and schizophrenic patients [32]. Considering their role in the formation, trafficking, and stabilization of NMDARs and AMPARs at excitatory synapses [33, 34], we can hypothesize that ketamine-induced increase of scaffolding protein in PSD herein observed is probably involved in a cortical increased trafficking and stabilization of glutamate receptors that might underlie ketamine antidepressant effects.

To note, the decrease of the glutamate transporter GLT-1 after ketamine in mPFC could be referred to different factors. In fact, the decrease of GLT-1, and thus the increase of glutamate synaptic concentration, has been linked to the development and maintenance of abuse to different substances, such as cocaine, nicotine, and ethanol, and the increase of GLT-1 in mPFC and nucleus accumbens was shown to correlate to relapse inhibition [35–37]. Thus, this increase could be a metaplastic effect of ketamine addictive properties lasting longer after acute administration. Nevertheless, this could as well be a sign of its antidepressant action, since recent reports demonstrated that glial GLT-1 blockade in mPFC contributes to the rapid onset of ketamine antidepressant effects [38, 39] and the astrocytic GLT-1 deficiency correlates to decreased anxiety and depression in a mouse model [40].

The glutamatergic modulation induced by MK-801 in the mPFC was different compared to ketamine, and few studies have specifically investigated the level of GluN1 and GluN2A subunits as well as scaffolding proteins after acute MK-801 in mPFC [41, 42]. To note, Xi and colleagues reported a significant downregulation of NMDARs subunits expressing-mRNA transcripts and protein levels in both pyramidal cells and parvalbumin-containing interneurons after acute high doses of MK-801 [41], a mechanism that could underlie the schizophrenia-like symptoms usually observed after acute or chronic administration.

### The Effects of Ketamine and MK-801 in the Hippocampus

In the Hipp, while MK-801-induced reduction of NMDARs was limited to the homogenate, ketamine decreased NMDARs also in the post-synaptic density, an effect that could be hypothesized to be related to a finer tuning of glutamatergic synapses by ketamine compared to MK-801. Previous research has reported a sustained decrease of GluN1 as well as a rapid but transient decrease of GluN2A and GluN2B as a molecular correlate of ketamine antidepressant action in Hipp [43] and of other antidepressant drugs action on stress-induced receptors upregulation [44]. On the contrary, Burgdorf and colleagues reported an increase of GluN2B (and GluA1) after 10 mg/kg i.v. ketamine in the cell surface of Hipp [10]. These discrepancies could be related to the different routes of administration, doses (30 mg/kg i.p. ketamine for Xia et al.), or cellular compartments analyzed, but further studies are needed to disambiguate these differences.

As far as concern AMPARs subunits, GluA1 was decreased at post-synaptic level by both compounds, with no changes in the homogenate. Moreover, while ketamine decreased GluA2 in the homogenate and in the post-synaptic density, MK-801 increased GluA2 in the post-synaptic density. Previous evidence indicates AMPARs activation and GluA1 increase in Hipp as fundamental for ketamine antidepressant action, likely through an mTOR activation-dependent mechanism [45–47]. In contrast, here, we observed the decrease of GluA1 after both NMDARs antagonists, similarly to the addiction-related effects exerted by ketamine long-term self-administration we previously reported [14].

Finally, the finer tuning of glutamate synapses by ketamine compared to MK-801 is further suggested by the results on scaffolding proteins. Ketamine reduced PSD95, SAP102, and SAP97 only in the post-synaptic density, while MK-801 decreased scaffolding proteins both in the homogenate and in the post-synaptic density. These neuroplastic changes could reflect an enhanced membrane receptors instability as well as a reduction of spine density or spine head volume, suggesting an ambiguous morphological and functional synaptic alteration after acute NMDARs antagonism, a pattern of modification that is more likely referable to a profile of mood disorders [48, 49]. In particular, the decrease of PSD95 in the post-synaptic density of hippocampus after ketamine is indeed more difficult to interpret, with respect to the available literature. In fact, in disagreement with our results, ketamine is usually applied to reverse PSD95 reduction induced by stressful conditions [30, 31].

### Metaplastic Effects of NMDARs Antagonists in Medial Prefrontal Cortex and Hippocampus

By a metaplasticity standpoint, it appears that ketamine induced a global change of all the components of glutamatergic transmission, while MK-801 changes were sparser and more limited to some subunits and scaffolding proteins in mPFC. It is important to note that the ketamine-induced increase of GluN2B and AMPARs subunits observed in the mPFC closely resembles the receptor changes observed after acute and repetitive substance abuse and withdrawal in the ventral tegmental area and in the nucleus accumbens [50, 51]. However, the glutamatergic changes reported here in mPFC are in contrast to the self-administered ketamine effects we previously observed [14, 15], suggesting a differential modulation of the glutamatergic tone by “antidepressant” vs “addictive” ketamine dose regimen. Moreover, considering the GluN2A/GluN2B ratio as an index of metaplasticity [8], we can conclude that both ketamine and MK-801 induced metaplastic effects, as both the compounds decrease the ratio thus determining a relative increase of the more Ca^2+^-permeable NMDAR subunit GluN2B in the mPFC, respectively in the total homogenate and in the post-synaptic density.

In Hipp, we observed a ketamine-induced modulation of AMPAR subunits resulting in a relative increase of GluA1/GluA2 functional ratio. This increase, in parallel with a relative decrease of GluN2A/GluN2B ratio, points to a general metaplastic enhancement of synaptic activity in the Hipp after ketamine, mediated by a relative heightening of calcium influx due to increased GluA1 and GluN2B relative levels. On the other hand, the opposite modulation of AMPARs subunits by MK-801, resulting in a decrease of GluA1/GluA2 ratio, most likely impairs hippocampal activity, presumably leading to a synaptic “crystallization” characterized by less susceptibility to structural and functional modification.

In conclusion, our study showed structural changes of glutamatergic synapses induced by the NMDARs antagonists MK-801 and ketamine that we could define as metaplastic. We hypothesize that the cascade of glutamatergic changes induced under this mode of dosing might affect synaptic efficiency and its modification by subsequent stimulations (e.g., tetanus, stressful conditions, and memory reactivation). Indeed, the same NMDARs antagonists can exert both protective and harmful effects on subjects suffering from mood disorders: the former, by ameliorating depressive symptoms in depressed patients, and the latter, by inducing or exacerbating psychotic symptoms. The state dependency of NMDARs antagonism final outcome is in line with the definition of ketamine antidepressant effects as metaplastic phenomena in clinical and preclinical evidence. In fact, depending on the basal state of glutamatergic synapses and on the therapeutic intervention applied closely to the treatment, ketamine and, to a lesser extent, MK-801 could act as reliable pharmacological strategies against mood disorders, such as depression or addiction. Considering the versatility action of NMDARs antagonism that could mediate addictive as well as therapeutic effects, further efforts are needed to clearly disambiguate the molecular and behavioral processes underpinning these metaplastic properties.

## Supplementary Information

ESM 1 (PDF 6506 kb)

## Data Availability

The authors confirm that the data supporting the findings of this study are available within the article and its supplementary materials. The raw data are available from Lucia Caffino (lucia.caffino@unimi.it) upon reasonable request.
