# Supplementary material for: Metaplastic Effects of Ketamine and MK-801 on Glutamate Receptors Expression in Rat Medial Prefrontal Cortex and Hippocampus
Source: Mol Neurobiol. 2021 Mar 15;58(7):3443–56. doi: 10.1007/s12035-021-02352-7 (PMC8257545; doi:10.1007/s12035-021-02352-7)

Supplementary figure 1. Uncropped immunoblot related to the expression levels of GluN2A (180 kDa), GluN2B (180 kDa), GluN1 (120 kDa), GluA1 (108 kDa), GluA2 (108 kDa), SAP102 (102 kDa), SAP97 (97 kDa), PSD95 (95 kDa), GLT-1 (62 kDa), β-actin (43 kDa) measured in the whole homogenate of medial prefrontal cortex of male rats exposed to an acute injection of saline, MK-801 or ketamine, presented in Figures 1, 2, 3, 4.

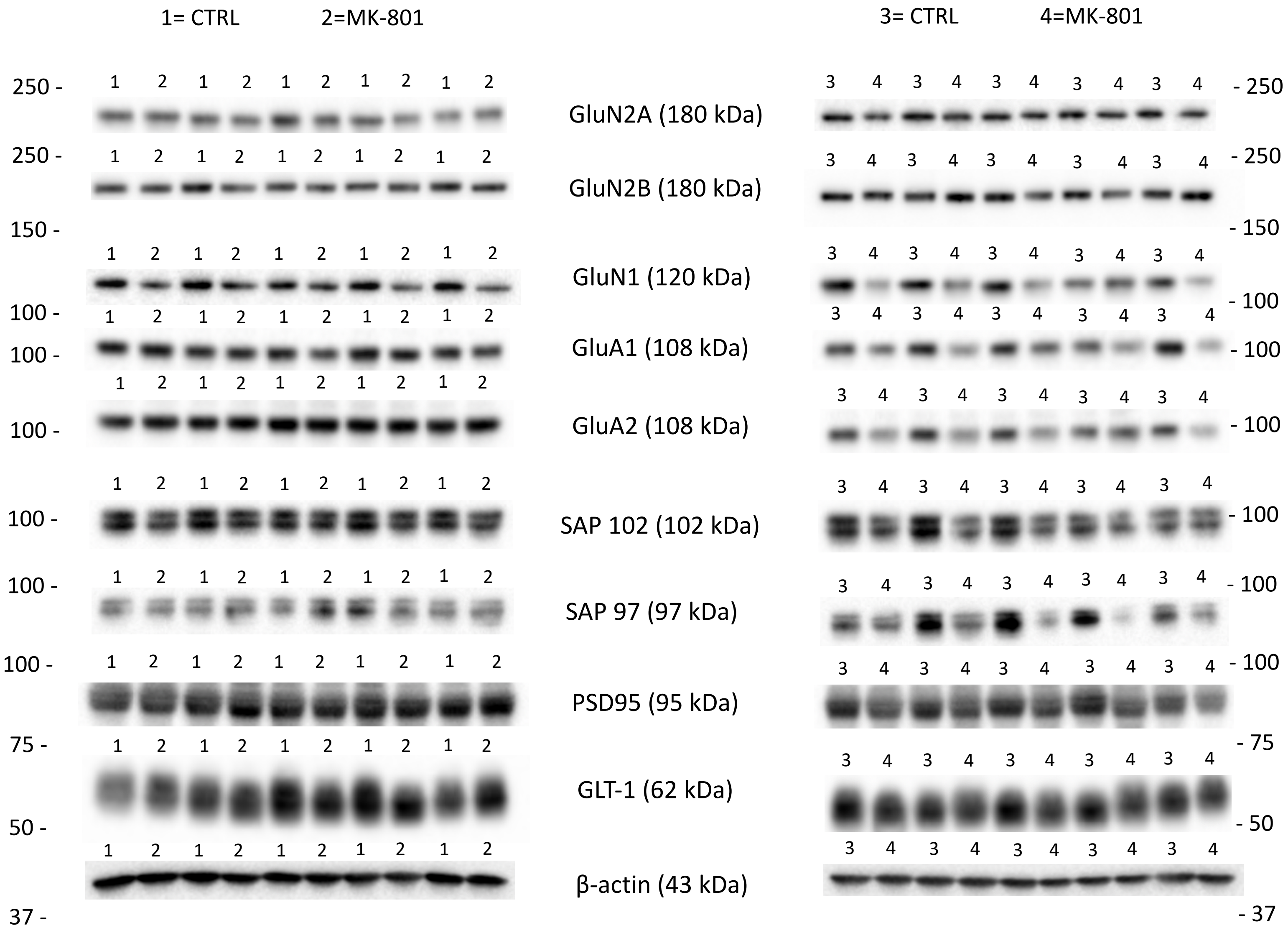

Supplementary figure 2. Uncropped immunoblot related to the expression levels of GluN2A (180 kDa), GluN2B (180 kDa), GluN1 (120 kDa), GluA1 (108 kDa), GluA2 (108 kDa), SAP102 (102 kDa), SAP97 (97 kDa), PSD95 (95 kDa), β-actin (43 kDa) measured in the post-synaptic density fraction of medial prefrontal cortex of male rats exposed to an acute injection of saline, MK-801 or ketamine, presented in Figures 1, 2, 3, 4.

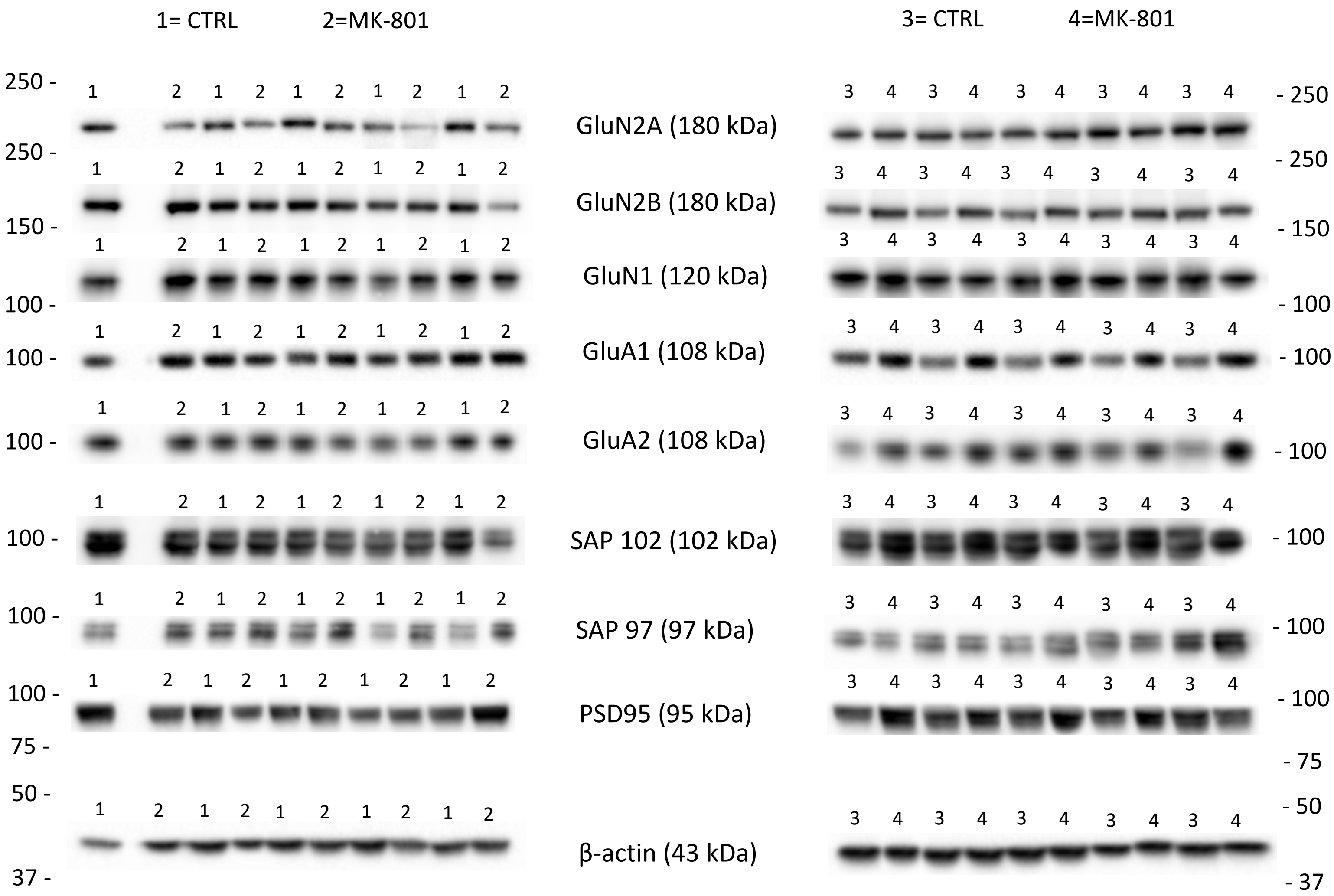

Supplementary figure 3. Uncropped immunoblot related to the expression levels of GluN2A (180 kDa), GluN2B (180 kDa), GluN1 (120 kDa), GluA1 (108 kDa), GluA2 (108 kDa), SAP102 (102 kDa), SAP97 (97 kDa), PSD95 (95 kDa), GLT-1 (62 kDa), β-actin (43 kDa) measured in the whole homogenate of hippocampus of male rats exposed to an acute injection of saline, MK-801 or ketamine, presented in Figures 6, 7, 8, 9.

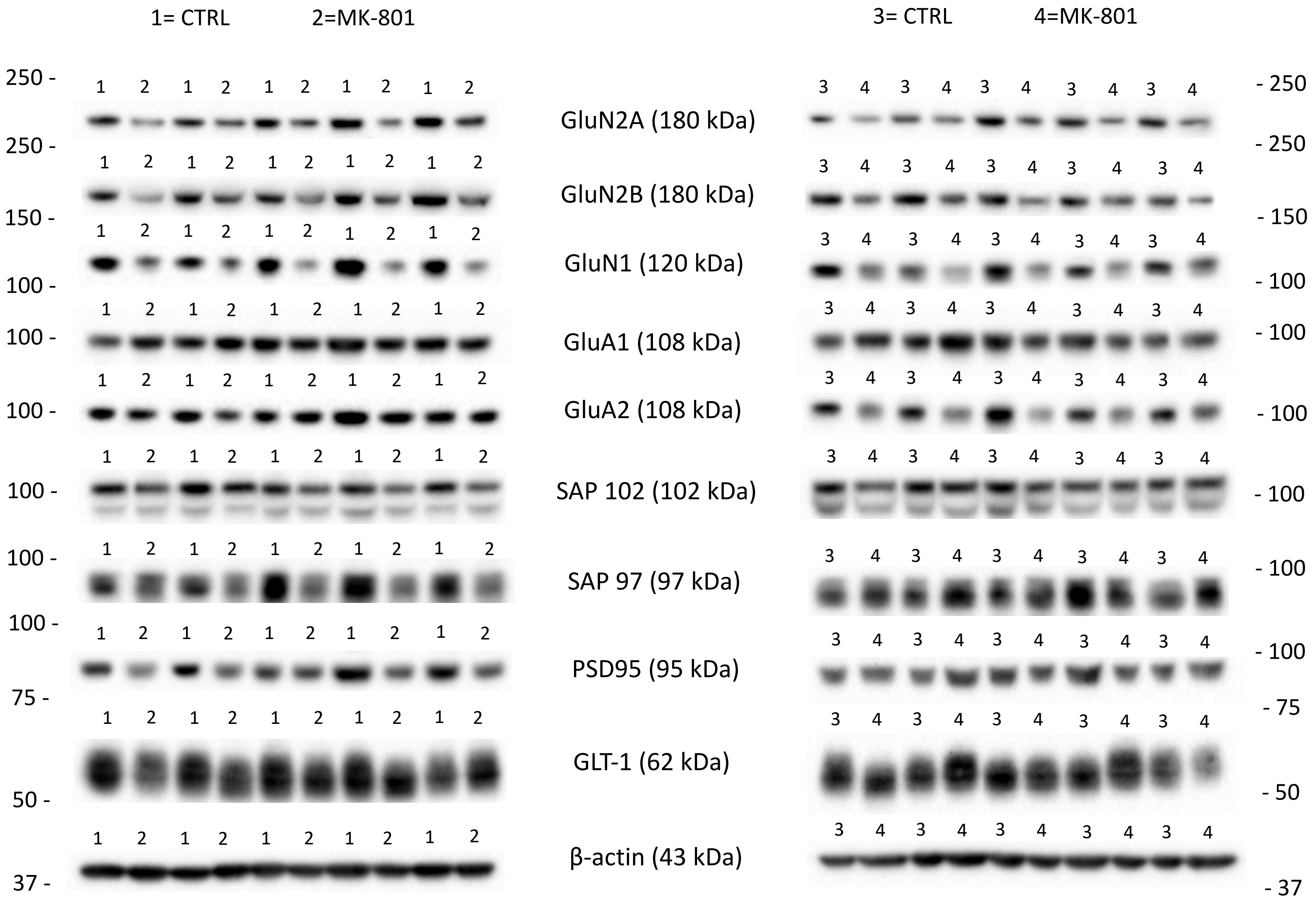

Supplementary figure 4. Uncropped immunoblot related to the expression levels of GluN2A (180 kDa), GluN2B (180 kDa), GluN1 (120 kDa), GluA1 (108 kDa), GluA2 (108 kDa), SAP102 (102 kDa), SAP97 (97 kDa), PSD95 (95 kDa), β-actin (43 kDa) measured in the post-synaptic density fraction of hippocampus of male rats exposed to an acute injection of saline, MK-801 or ketamine, presented in Figures 6, 7, 8, 9.

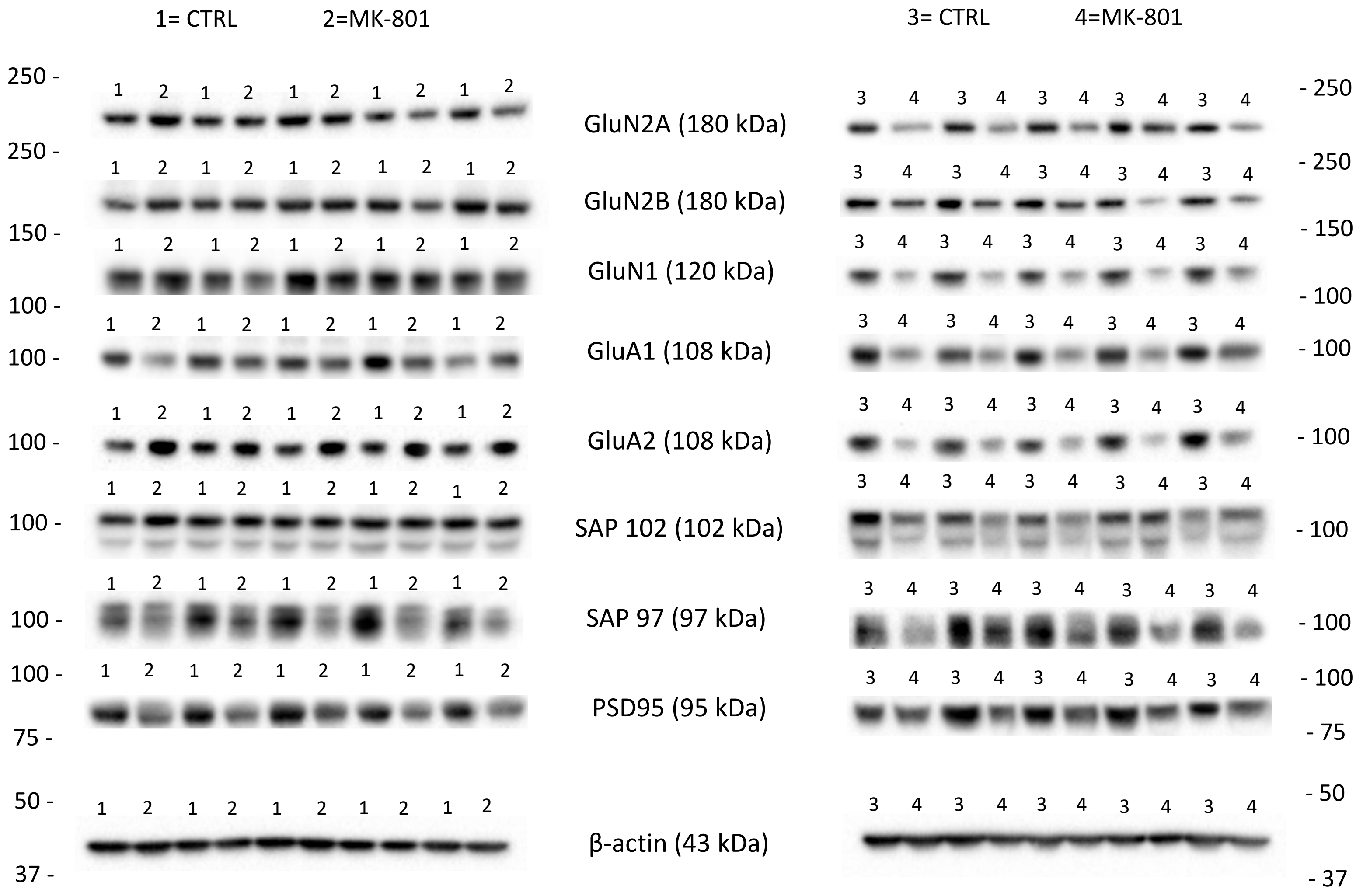

Supplement: Supplementary file 1 — (PDF 6506 kb) [file 12035_2021_2352_MOESM1_ESM.pdf]
